# Moroccans’ Perception of Addiction: A Cross-Sectional Study on Stigma and Familiarity Dynamics

**DOI:** 10.1192/bjo.2022.224

**Published:** 2022-06-20

**Authors:** Ibrahim M'hamdi, Afaf Harbil, Abdessamad Bensaid, Omar Benchekroun

**Affiliations:** 1 I. Horbachevsky Ternopil National Medical University, Ternopil, Ukraine; 2 Faculty of Medicine and Pharmacy of Fez, Sidi Mohamed Ben Abdellah University, Fez, Morocco; 3 KTH Royal Institute of Technology, Stockholm, Sweden; 4 Grenoble-INP ENSIMAG, Grenoble, France

## Abstract

**Aims:**

This study aims to assess the stigmatization of Moroccans towards substance and nonsubstance addictions, as well as to explore its relationship with both demographic factors and addiction familiarity.

**Methods:**

527 Moroccans anonymously participated in a cross-sectional study via an online survey that was distributed on social media. Participants were randomly assigned 2 vignettes describing either substance (Alcohol and Cannabis) or non-substance (Gambling and Social Media) addictions, followed by the Social Distance Scale and the Familiarity Scale.

**Results:**

A total of 527 individuals answered our online questionnaire. The median age of respondents was 27.6 years (std = 15.66). 56% were females and 44% were males. Among the participants 45% were married and 50% were medical students or health professionals.

Using ANOVA and a series of student t-tests, that yielded a p < 0.05, the following results were obtained:

A moderate level of stigma was found towards all addictions, except for social media where no stigma was found (p < 0.05). In contrast, the familiarity level was high with social media addiction and low with the other addictions (p < 0.05).

The women in our study showed higher stigmatization of all addictions, whereas older people (>43 years) showed higher stigmatization of substance addictions only.

Different levels of stigmatization were observed towards the 4 types of addiction; the highest being cannabis addiction and the lowest being social media addiction.

Regarding familiarity with addiction, males were more familiar with all types of addiction. Whereas, younger individuals (<23 years) were the least familiar with substance addiction.

Moroccans’ familiarity levels with different types of addiction were significantly different. Familiarity with social media addiction was the highest whereas familiarity with gambling addiction was the lowest.

Using the Pearson correlation, we found that stigma and familiarity concerning substance addiction were negatively correlated (r=−0,30, p < 0.01). A stronger, yet moderate relationship was found between stigma and familiarity regarding cannabis (r=−0,36, p < 0.01).

**Conclusion:**

It seems that Moroccans stigmatize against most addictions, which was found to be influenced by multiple factors including familiarity level, age, and sex. These findings can be used as a base to create a targeted educational campaign to tackle addiction in our society. No significant conclusions were made concerning whether or not the academic level or the health professional background influenced stigmatization, which raises concerns about the Moroccan academic and medical curricula's representation of addiction.

